# Current mentorship practices in the training of the next generation of clinical microbiology and infectious disease specialists: an international cross-sectional survey

**DOI:** 10.1007/s10096-019-03509-y

**Published:** 2019-02-19

**Authors:** David S. Y. Ong, Thea Christine Zapf, Muge Cevik, Zaira R. Palacios-Baena, Aleksandra Barać, Cansu Cimen, Alberto E. Maraolo, Caroline Rönnberg, Emmanuelle Cambau, Mario Poljak, On behalf of the Trainee Association of the European Society of Clinical Microbiology and Infectious Diseases (ESCMID)

**Affiliations:** 1 Department of Medical Microbiology and Infection Control, Franciscus Gasthuis & Vlietland, Rotterdam, The Netherlands; 2 Department of Epidemiology, Julius Center for Health Sciences and Primary Care, University Medical Center Utrecht, Utrecht, The Netherlands; 3 Department of Medical Microbiology and Infection Prevention, University Hospital of Marburg, Marburg, Germany; 4 Division of Infection and Global Health Research, School of Medicine, University of St Andrews, St Andrews, UK; 5 Regional Infectious Diseases Unit, Western General Hospital, Edinburgh, UK; 6 Department of Laboratory Medicine, Royal Infirmary of Edinburgh, Edinburgh, UK; 7 Unidad de Gestión Clínica de Enfermedades Infecciosas y Microbiología / Instituto de Biomedicina de Sevilla (IBIS), Hospital Universitario Virgen Macarena / Universidad de Sevilla, Seville, Spain; 8 Clinic for Infectious and Tropical Diseases, Clinical Centre of Serbia, Belgrade, Serbia; 9 Faculty of Medicine, University of Belgrade, Belgrade, Serbia; 10 Infectious Diseases and Clinical Microbiology Clinic, Ministry of Health Ardahan Public Hospital, Ardahan, Turkey; 11 Department of Clinical Medicine and Surgery, Section of Infectious Diseases, University of Naples Federico II, Naples, Italy; 12 Department of Clinical Microbiology, Karolinska University Hospital, Stockholm, Sweden; 13 Clinical Microbiology Laboratory, APHP-Lariboisiere Hospital, Paris, France; 14 School of Medicine, University of Paris Diderot, UMR1137 IAME, Paris, France; 15 Institute of Microbiology and Immunology, Faculty of Medicine, University of Ljubljana, Ljubljana, Slovenia

**Keywords:** Mentorship, Education, Training, Clinical microbiology, Infectious diseases, Mentoring

## Abstract

The purpose of this study was to describe the current practice of mentorship in clinical microbiology (CM) and infectious diseases (ID) training, to identify possible areas for improvement and to assess the factors that are associated with satisfactory mentorship. An international cross-sectional survey containing 35 questions was answered by 317 trainees or specialists who recently completed clinical training. Overall, 179/317 (56%) trainees were satisfied with their mentors, ranging from 7/9 (78%) in non-European countries, 39/53 (74%) in Northern Europe, 13/22 (59%) in Eastern Europe, 61/110 (56%) in Western Europe, 37/76 (49%) in South-Western Europe to 22/47 (47%) in South-Eastern Europe. However, only 115/317 (36%) respondents stated that they were assigned an official mentor during their training. In multivariable logistic regression analysis, the satisfaction of trainees was significantly associated with having a mentor who was a career model (OR 6.4, 95%CI 3.5–11.7), gave constructive feedback on work performance (OR 3.3, 95%CI 1.8–6.2), and knew the family structure of the mentee (OR 5.5, 95%CI 3.0–10.1). If trainees felt overburdened, 70/317 (22%) felt that they could not talk to their mentors. Moreover, 67/317 (21%) stated that they could not talk to their mentor when unfairly treated and 59/317 (19%) felt uncertain. Training boards and authorities responsible for developing and monitoring CM&ID training programmes should invest in the development of high-quality mentorship programmes for trainees in order to contribute to the careers of the next generation of professionals.

**Electronic supplementary material:**

The online version of this article (10.1007/s10096-019-03509-y) contains supplementary material, which is available to authorized users.

## Introduction

Mentorship is a professional interaction that occurs between two people, i.e. mentor and mentee, of different levels of knowledge and expertise [1]. The mentors act as a role model relaying essential knowledge to the mentee not only on career progression but also on professionalism, ethics and personal development [2, 3]. Mentorship is recognised as a fundamental tool for professional development, and the presence of a mentor has been associated with positive training outcomes, greater career satisfaction and better work-life balance of mentees [4–6]. In fact, it is considered one of the most critical determinants of career success in medicine and research [7, 8].

Mentors are highly valuable to help shape the careers of the next generation of medical specialists who need to acquire both medical and professional knowledge and skills in a limited period of time. At the same time, medical professionals in training may be confronted with uncertainties and challenges because of inexperience, strong dependence on superiors, temporary work contracts, and sometimes sudden and unexpected major changes in personal life and working conditions. However, there is no well-established framework to implement a mentoring scheme in clinical training. In an era of rapid evolution of medical and technological opportunities, the bar of learning is set much higher for trainees of today. This particularly stands as a significant challenge for trainees specialising in clinical microbiology (CM) or infectious diseases (ID) [9]. Although the majority of CM&ID trainees in Europe expressed the desire to receive more mentorship during their training [10], current mentorship practices in these specialties and how these are perceived by trainees are mostly unknown.

This study aimed to describe for the first time the current practice of mentorship in CM&ID training in Europe, especially, to identify possible areas for improvement and to assess factors that are associated with satisfactory mentorship.

## Methods

### Study design

The survey was conceived in 2017 by the Steering Committee of the Trainee Association of the European Society of Clinical Microbiology and Infectious Diseases (TAE). The study included survey responses from trainees in CM&ID or young CM&ID specialists within 3 years after completion of training.

Between the 19th of January and the 9th of February 2017, a pilot survey was performed among 36 trainees in Germany, Italy, the Netherlands, Spain, Sweden, and Turkey. Following the feedback received on the pilot study, several amendments were made. The final survey was published online on an open source SoSci survey platform (version 2.5.00-i, SoSci Survey GmbH, Munich, Germany) between the 1st of June and the 30th of September 2017. The survey was distributed among trainees in Europe using the TAE communication network, which included representatives of CM&ID national societies from 37 out of 58 (64%) European countries. The survey was also promoted through the TAE website (i.e., www.escmid.org/tae), social media accounts, and the ESCMID monthly newsletters, received by more than 25,000 professionals. At the beginning of the survey, the respondents were informed about the purpose of the study and the anonymity of survey results and that no financial or other compensation for participation in this survey would be provided.

The survey included 19 sociodemographic questions and 16 questions on mentorship (eTable 1). In the survey, a mentor was defined as an experienced person who should provide help and guidance to a less experienced person during their training period. A mentor may give psychosocial support, career guidance, role modelling, and/or informal communication usually face-to-face and during a sustained period of time. When there was not a mentor officially assigned to the trainee, respondents were asked to fill in the survey keeping in mind a person who has guided them the most during the training period.

### Data analysis

Categorical variables were summarised by frequencies and percentages. Continuous data were presented as medians with interquartile range (IQR). We compared groups using nonparametric tests for continuous variables and chi-square test for categorical variables. To assess regional differences, the countries of participants were categorised into five categories used regularly by the European Society of Clinical Microbiology and Infectious Diseases (ESCMID) and a sixth category of non-European countries (eTable 2). In univariable analyses we assessed which sociodemographic and mentorship characteristics were associated with mentorship satisfaction. Multivariable logistic regression analysis was performed, which included only the factors with a *p* value < 0.20 according to univariable analyses. *p* values < 0.05 were considered to be statistically significant. All analyses were performed using SAS 9.2 (Cary, NC, USA) and R version 3.3.2 (R Foundation for Statistical Computing).

## Results

### Sociodemographic data

Responses from 356 survey participants were received. After exclusion of medical specialists who had finished their training more than 3 years ago, a total of 317 participants were included in the analysis (Table 1). The median age of participants was 32 years (IQR 30–35 years). Most respondents were female (*n* = 196, 62%) and 71 (22%) were recently certified medical specialists. The majority of respondents were working in Western Europe (*n* = 110, 35%) and South-Western Europe (*n* = 76, 24%), followed by Northern Europe (*n* = 53, 17%), South-Eastern Europe (*n* = 47, 15%), Eastern Europe (*n* = 22, 7%), and outside of Europe (*n* = 9, 3%). One hundred twenty-nine respondents were trainee or specialist in CM, 146 in ID, and 42 were in both CM&ID.

**Table 1:** General characteristics of respondents

Characteristics	All (*n* = 317)	Western Europe (*n* = 110)	Northern Europe (*n* = 53)	Eastern Europe (*n* = 22)	South-Western Europe (*n* = 76)	South-Eastern Europe (*n* = 47)	Non-European countries (*n* = 9) ^b^	*p* value ^c^
Age (years)	32 (30–35)	32 (30–35)	35 (32–39)	29 (27–30)	30 (28–31.5)	33 (31–39)	33 (33–39)	< 0.001
Female gender ^a^	196 (62)	65 (60)	32 (60)	13 (59)	44 (58)	38 (81)	4 (44)	0.101
Marital status (married or living with a partner)	195 (62)	77 (71)	40 (75)	12 (55)	29 (38)	32 (68)	5 (56)	< 0.001
Parental status (parent)	102 (32)	39 (36)	36 (68)	2 (9)	3 (4)	19 (40)	3 (33)	< 0.001
Training status (trainee, i.e. not medical specialist)	246 (78)	99 (91)	45 (85)	18 (82)	54 (71)	24 (51)	5 (56)	< 0.001
Specialty								< 0.001
CM	129 (41)	49 (45)	27 (51)	2 (9)	28 (37)	20 (43)	2 (22)	
ID	146 (46)	41 (38)	23 (43)	20 (91)	47 (62)	9 (19)	6 (67)	
CM/ID	42 (13)	19 (17)	3 (6)	0 (0)	1 (1)	18 (38)	1 (11)	
More than 20 overtime hours per month	109 (34)	38 (35)	3 (6)	11 (50)	37 (49)	20 (43)	0 (0)	< 0.001

Data are presented in median (interquartile range) or in absolute number (percentage)

^a^ 1 participant responded non-binary

^b^ Brazil (*n* = 1), Iran (*n* = 1), Israel (*n* = 2), Kenya (*n* = 1), Nigeria (*n* = 1), Peru (*n* = 1), the USA (*n* = 2)

^c^ Kruskal-Wallis test for continuous variables and chi-square for categorical variables

Looking at regional differences within Europe (Table 1), the median age was highest in participants from Northern Europe (35 years, IQR 32–39) and lowest in Eastern (29, IQR 27–30) and South-Western Europe (30, IQR 28–31.5) (*p* < 0.001). Marital status was also different between regions; the highest proportion of respondents who were married or living in a stable partnership was from Northern Europe (*n* = 40, 75%) and the lowest from South-Western Europe (*n* = 29, 38%) (*p* < 0.001). Similarly, parental status (i.e., whether or not a person is a parent) was highest for Northern Europe (*n* = 36, 68%) and lowest for South-Western Europe (*n* = 3, 4%) (*p* < 0.001).

### Mentor-mentee relationship

Only 115/317 (36%) respondents stated that they were assigned an official mentor during their training, whereas the remaining 202/317 (64%) respondents had no official mentors (Table 2). Most of the mentors (i.e., 266/317 (84%)) were from the same medical specialty as the respondent. Moreover, 185/317 (58%) respondents considered their mentors to be a career model (i.e., role model to which the mentee looks up career-wise), while 159/317 (50%) respondents stated that their mentors contributed to shaping their careers. Up to two-thirds of mentors were involved in the daily work of respondents (*n* = 210, 66%) or gave constructive feedback on their work (*n* = 189, 60%).

**Table 2:** Mentorship practice according to region

Characteristics	All *n* = 317 (100%)	Western Europe *n* = 110 (35%)	Northern Europe *n* = 53 (17%)	Eastern Europe *n* = 22 (7%)	South-Western Europe *n* = 76 (24%)	South-Eastern Europe *n* = 47 (15%)	Non-European countries *n* = 9 (3%)	*p* value
Assigned to mentor	115 (36)	21 (19)	23 (43)	8 (36)	32 (42)	26 (55)	5 (56)	< 0.001
Possibility to choose mentor (instead of being assigned to one)	81 (26)	32 (29)	14 (26)	8 (36)	9 (12)	11 (23)	6 (67)	0.003
Mentor is from the same medical specialty as the respondent	266 (84)	92 (84)	47 (89)	21 (95)	64 (84)	35 (74)	6 (67)	0.146
Mentor is a career model	185 (58)	58 (53)	35 (66)	14 (63)	47 (62)	23 (49)	7 (78)	0.302
Mentor gives information about how to shape the career of the respondent	159 (50)	60 (55)	30 (57)	10 (45)	33 (43)	19 (40)	6 (67)	0.265
Mentor is involved in daily work of respondent at least several times per month	210 (66)	71 (65)	44 (83)	18 (82)	46 (61)	25 (53)	5 (56)	0.014
Mentor gives constructive feedback on the work of the respondent	189 (60)	67 (61)	35 (66)	17 (77)	40 (53)	21 (45)	8 (89)	0.023
Mentors are trusted to be confidential	230 (73)	87 (80)	44 (83)	17 (77)	46 (61)	30 (64)	5 (56)	0.011
Mentor is working for the same boss as respondent								< 0.001
Yes	61 (19)	18 (17)	5 (9)	7 (32)	10 (13)	17 (36)	4 (44)	
No	213 (67)	76 (68)	45 (85)	12 (55)	58 (76)	19 (40)	3 (33)	
I do not know	43 (13)	15 (14)	3 (6)	3 (14)	8 (11)	11 (23)	2 (22)	
Possibility of talking to mentor when feeling overburdened								0.002
Yes	195 (62)	70 (64)	41 (77)	13 (59)	42 (55)	25 (53)	4 (44)	
No	70 (22)	18 (17)	3 (6)	5 (23)	29 (38)	12 (26)	3 (33)	
I do not know	52 (16)	21 (19)	9 (17)	4 (18)	5 (7)	10 (21)	2 (22)	
Possibility of talking to mentor when unfairly treated								0.019
Yes	191 (60)	72 (66)	35 (66)	12 (55)	41 (54)	26 (55)	5 (56)	
No	67 (21)	18 (17)	5 (9)	5 (23)	28 (37)	9 (19)	2 (22)	
I do not know	59 (19)	19 (17)	13 (25)	5 (23)	7 (9)	12 (26)	2 (22)	
Possibility of talking to mentor when experiencing problems with main supervisor								0.091
Yes	170 (54)	58 (53)	33 (62)	11 (50)	40 (53)	22 (47)	6 (67)	
No	91 (29)	26 (24)	12 (23)	6 (27)	31 (41)	14 (30)	2 (22)	
I do not know	56 (18)	25 (23)	8 (15)	5 (23)	5 (7)	11 (23)	1 (11)	
Mentor knows the family structure of respondent								0.144
Yes	183 (58)	65 (60)	39 (74)	13 (59)	39 (51)	23 (49)	4 (44)	
No	85 (27)	24 (22)	9 (17)	5 (23)	28 (37)	15 (32)	4 (44)	
I do not know	49 (15)	20 (18)	5 (9)	4 (18)	9 (12)	9 (19)	1 (11)	
Respondent is satisfied about mentor	179 (56)	61 (56)	39 (74)	13 (59)	37 (49)	22 (47)	7 (78)	0.039

Data are presented in absolute number (percentage)

Furthermore, 230/317 (73%) trainees trusted their mentor to maintain confidentiality; however, heterogeneity between regions was observed ranging from 44/53 (83%) in Northern Europe to 46/76 (61%) in South-Western Europe (Table 2). In the majority of cases, the mentors and mentees were working under different superiors. If trainees felt overburdened, 195/317 (62%) felt that they could talk to their mentors, 70/317 (22%) felt they could not talk and 52/317 (16%) were uncertain. Moreover, 67/317 (21%) of the respondents stated that they could not talk to their mentor if unfairly treated at work and 59/317 (19%) felt uncertain about it. Mentees stated that their mentors were aware of their family structure in 183/317 (58%) of the cases.

### Satisfaction of the mentee with mentorship

Overall, 179/317 (56%) of trainees were satisfied with their mentors ranging from 39/53 (74%) in Northern to 22/47 (47%) in South-Eastern Europe (Table 2). In a univariable analysis, age, gender, marital status, parenteral status, training status, and working more than 20 overtime hours per month were not associated with the satisfaction of the mentee regarding the mentorship received; therefore, these variables were not included in the multivariable analysis. In the final multivariable model, the satisfaction of the mentee was independently associated with having a mentor who was a career model for the trainee, gave constructive feedback on their performance, and knew the family structure of the mentee (Table 3). However, the satisfaction of the mentee was not associated with mentor and mentee having the same medical speciality background (*p* = 0.82), the involvement of mentor in the daily work of mentee (*p* = 0.08), and mentor and mentee having different superiors (*p* = 0.75).

**Table 3:** Factors associated with mentee’s satisfaction with mentorship

Factors	Univariable analysis OR (95% confidence interval)	Multivariable analysis OR (95% confidence interval)	*p* value (for multivariable model)
Mentor is from the same medical specialty	4.3 (2.2–8.4)	0.9 (0.3–2.3)	0.81
Mentor is a career model	8.5 (5.1–14.1)	6.4 (3.5–11.7)	< 0.01
Mentor is involved in daily work of respondent several times per month	2.9 (1.8–4.7)	1.7 (0.9–3.3)	0.08
Mentor gives constructive feedback on the work of the respondent	6.5 (4.0–10.7)	3.3 (1.8–6.2)	< 0.01
Mentor is working under the same superior as respondent	1.5 (0.8–2.6)	0.9 (0.4–1.8)	0.75
Mentor knows the family structure of respondent	7.0 (4.2–11.4)	5.5 (3.0–10.1)	< 0.01

## Discussion

The present study demonstrates that only one-third of CM&ID trainees in Europe had been officially assigned a mentor during their clinical training. Although the majority of the mentors were from the same medical specialty as the mentee, mentors were a career model for only 58% of the respondents and only half of the mentees have felt that mentors significantly contributed to shaping their career. Despite that, 56% of respondents were satisfied with the way they were mentored during training. In multivariable analysis, the satisfaction of trainees was significantly associated with having a mentor who was a career model, gave constructive feedback on the work performance, and knew the family structure of the mentee.

Thriving mentorship is known to be crucial for career success [7]. Irrespective of the different possible roles a mentor could fulfil [11], the mentor is expected to be present and available for the mentee and have the necessary professional and scientific skills to guide the mentee [9]. Good mentors should understand mentees’ needs and be able to listen actively and be approachable, and their communication with mentees should be sincere and confidential [12]. However, according to our survey, more than a quarter of respondents did not consider their relationships with mentors to be confidential and just over half felt comfortable approaching their mentor when they feel overburdened or unfairly treated at work. Confidentiality and sincere communication are necessary interpersonal abilities in any mentorship practice [13]. Furthermore, characteristics of mentorship malpractice including hijack (i.e., taking mentee’s ideas or projects and labelling them as his or her own for self-gain), exploitation, and possessive behaviour of mentors are evident barriers to fruitful mentorship [14].

Low-quality mentorship is not only observed in CM&ID training but also broadly recognised among other medical specialties as well as in academic medicine [5–7, 12, 13, 15–17]. Barriers to mentoring and inadequate mentoring can be related to many factors including personal, relational, and structural barriers [12]. Most of the evidence suggests that mentoring provides a consistent benefit for both the mentor and the mentee when mentors are selected well according to their personality and attributes, and are trained in the required skills [18]. Although some senior academics and medical specialists are excellent mentors by nature, structured mentorship training may optimise the benefit and satisfaction of mentee-mentor relationships. Such training programmes focusing on promoting the characteristics of good and effective mentorship (sharing mutual goals, respect, trust) as well as the responsibilities of mentees and mentors are needed [13, 19]. The ESCMID, one of the largest and most influential international societies in CM&ID, supports trainees and young scientists during their training by providing an opportunity to join its mentorship programme [20]. This programme would be beneficial for trainees as an additional opportunity to be mentored by experienced mentors from different institutions based on their area of interest and expertise.

According to a previous TAE survey, maintaining a good work-life balance has been identified as an essential aspect of training for CM&ID trainees [21]. The work-life balance could be supported through effective mentorship. It is important to state that effective mentorship is also dependent on the mentee’s skills. Golden rules for the mentee’s skills have been identified as the selection of the right mentor, maintaining respect, communicating effectively, and being engaged, energising, and collaborative [8, 19].

To the best of our knowledge, this is the first European study assessing the current mentorship practice among CM&ID trainees. We were able to collect data from all regions within Europe and provide an international overview of the current mentorship practice. We included respondents who recently have finished their specialist training (≤ 3 years), as past experiences may not be representative of the current situation and may lead to recall bias. Comparing mentorship characteristics and satisfaction of the mentee between the respondents who were still in training at the time of the survey to the respondents who were recently finished medical specialists did not show significant differences.

Our study has important limitations. First, we could not calculate the response rate of the survey as the total number of trainees in many countries is unknown. Second, Eastern Europe was underrepresented, although trainees and young medical specialists from that region have been actively contacted as well. Third, many respondents did not have an officially assigned mentor. In those instances, the questions were directed to consider a person who has guided them the most. Therefore, the mentors had varied backgrounds ranging from the heads of departments to supervisors and to senior residents. Most of the time, the supervisor and mentor were the same person, and this may potentially lead to conflicting interests. Mentorship is not intended to replace the supervisor role but aims to provide guidance of building a career as well as the prevention of burnout by striving for an adequate work-life balance [22]. Fourth, this survey focused primarily on the mentor and not on the mentee. Finally, in this survey, we estimated the mentorship of a single mentor. However, mentorship can also involve multiple mentors because a single person is unlikely to satisfy all mentoring needs of a trainee, and there is potentially a need for different mentors for different needs [14].

In conclusion, mentorship is often not available for CM&ID trainees in Europe and, when available, frequently lacks several characteristics required for adequate mentorship. Training boards and authorities responsible for developing and monitoring training programmes of CM&ID should invest in the development of high-quality mentorship programmes for trainees.

## Electronic supplementary material

ESM 1 (DOCX 16 kb)
