# Supplementary material for: Current mentorship practices in the training of the next generation of clinical microbiology and infectious disease specialists: an international cross-sectional survey
Source: Eur J Clin Microbiol Infect Dis. 2019 Feb 19;38(4):659–65. doi: 10.1007/s10096-019-03509-y (PMC6424943; doi:10.1007/s10096-019-03509-y)
Supplement: Supplementary file 1 — (DOCX 16 kb) [file 10096_2019_3509_MOESM1_ESM.docx]

**Supplementary material**

**eTable 1. Survey questions**

**Demographic questions:**

1. Age.
2. Gender
3. Marital status:
   1. Single
   2. In a relationship but not living together
   3. Not married but living with a partner
   4. Married
   5. Other
4. Do you have children? yes/no; How many?.
5. In which country do you work?
6. Is infectious diseases a recognized specialty in your country? Yes/no.
7. Is clinical microbiology a recognized specialty in your country? Yes/no.
8. Are you a trainee or a specialist in your field or work? Trainee/specialist.
9. Since how many years are you a specialist? Years since I finished my training.
10. Since how many years are you a trainee? Years since I started my training.
11. Did you have a written plan for your entire specialty training, when you were a trainee? Yes/no.
12. Did you have a written plan for your specialty? Yes/no.
13. What is your field of specialization? CM/ID/both: CM and ID/other.
14. Where do you primarily work?
    1. University hospital or large national hospital.
    2. Non-university hospital with more than 750 beds.
    3. Non-university hospital with less than 750 beds.
    4. Private clinic.
    5. Other, public health or research centre.
15. How many trainees are working in your department on the daily clinical work? (by counting, please include yourself in the total, if you are a trainee).
16. How many specialists are working in your department on the daily clinical work? (by counting, please include yourself in the total, if you are a specialist).
17. The workload in my field of specialization is acceptable for me. Please choose what applies best to you:
    1. Completely disagree
    2. Disagree
    3. Neither agree nor disagree
    4. Agree
    5. Completely agree
18. What is your average number of working hours per week?
    1. < 10 h per week.
    2. 10-20 h per week.
    3. 20-30 h per week.
    4. 30-40 h per week.
    5. >40 h per week.
19. What is your average number of working hours per month?
    1. < 10 overtime hours per month.
    2. 10-20 overtime hours per month.
    3. 20-30 overtime hours per month.
    4. >30 overtime hours per month.

**Mentorship questions:**

1. Have you been assigned a mentor?
2. Did you have the possibility to choose your mentor (as opposed to being assigned to one)?
3. Is your mentor a career-model for you?
4. Does your mentor give you information about how to shape your career?
5. Do you trust your mentor regarding confidentiality?
6. Is your mentor from the same specialty as yourself?
7. Does your mentor give you constructive feedback on your work?
8. Is your mentor “neutral” i.e. not working for the same boss as yourself?
9. Can you talk to your mentor if you feel overburdened?
10. Can you talk to your mentor if you feel unfairly treated?
11. Can you talk to your mentor about problems you are experiencing with your main supervisor?
12. Can you talk to your mentor and seek advice about personal issues?
13. Is your mentor aware of your family structure? (i.e. if you have children, does he/she help you e.g. with advice on keeping a work-life balance?)
14. How satisfied are you with your mentor? (scale 1 to 5; 1 = not satisfied at all, 2 = not satisfied, 3 = undetermined, 4 = satisfied, 5 = completely satisfied)
15. Is your mentor involved in your daily work? (scale 1 to 5; 1 = no, not at all, 2 = less then once a month, 3 = several times per month, 4 = several times per week, 5 = yes, we work together on a daily basis)
16. How often can you talk to your mentor? (scale 1 to 5; 1 = once or less than once per month, 2 = 2-3 times per month, 3 = once a week, 4 = 2-3 times per week, 5 = daily)

**eTable 2. ESCMID classification of European regions used in this study**

| **European region** | **Countries** |
| --- | --- |
| Western Europe | Austria, Belgium, France, Germany, Republic of Ireland, Liechtenstein, Luxembourg, The Netherlands, Switzerland, United Kingdom |
| Northern Europe | Denmark, Finland, Iceland, Norway, Sweden |
| Eastern Europe | Armenia, Azerbaijan, Belarus, Czech Republic, Estonia, Georgia, Hungary, Latvia, Lithuania, Moldova, Poland, Romania, Russia, Slovakia, Ukraine |
| South-Western Europe | Andorra, Italy, Malta, Monaco, Portugal, San Marino, Spain |
| South-Eastern Europe | Albania, Bosnia and Herzegovina, Bulgaria, Croatia, Cyprus, Greece, Israel, Kosovo, FYR of Macedonia, Montenegro, Serbia, Slovenia, Turkey |
